# Person-centred care and its outcomes in primary care. Selected Abstracts from the 96th EGPRN Meeting, Split–Croatia, 11–14 May 2023: *All abstracts of the conference can be found at the EGPRN website * https://www.egprn.org/page/conference-abstracts

**DOI:** 10.1080/13814788.2023.2248374

**Published:** 2023-08-24

**Authors:** Mine Kaya Bezgin

**Keywords:** Person centred care, long COVID, older people, type 2 diabetes melitus, colorectal cancer, LGBTIQ+, COVID-19

 

