# Supplementary material for: Person-centred care and its outcomes in primary care. Selected Abstracts from the 96th EGPRN Meeting, Split–Croatia, 11–14 May 2023: All abstracts of the conference can be found at the EGPRN website https://www.egprn.org/page/conference-abstracts
Source: Eur J Gen Pract. 2023 Aug 24;29(1):2248374. doi: 10.1080/13814788.2023.2248374 (PMC10453984; doi:10.1080/13814788.2023.2248374)
Supplement: Related texts

# Abstract: Introduction to the conference theme: ‘Person-centred care and its outcomes in primary care’

## Abstract

Person-centred care and its outcomes in primary care reflects the importance of applying person-centred care in everyday practice and measuring the objective outcomes of that care. Person-centred care (PCC) has been associated with many positive outcomes of care: patients’ emotional state, satisfaction and empowerment, medication adherence, reduction of malpractice, complaints and improvements in physician satisfaction and consultation time. Regardless of the specific context of care that is highly dependent on the patient, physician and healthcare system characteristics, PCC represents a core value of family medicine that should be implemented in family physicians’ everyday work across Europe. Yet despite growing recognition of the importance of PCC, as well as evidence of its effectiveness in contributing to other system goals such as efficiency and effectiveness, the nation’s health care system falls short of achieving it. Furthermore, data from national and international studies indicate that patients often rate hospitals and medical care providers highly but report significant problems in gaining access to critical information, understanding treatment options, getting explanations regarding medications, and receiving responsive, compassionate service from their healthcare professionals.

COVID-19 has challenged nearly everything about healthcare delivery, including the experiences of patients, families and healthcare professionals. Amid COVID-19, patient trust is on the line; virtual care has been embraced as never before. Nearly everything has become virtual; family visits over Skype, given visitation restrictions and barriers to virtual reimbursement and accessibility have come down, at least temporarily. Post COVID-19, with the virtual platforms and apps that have been stood up between clinicians and the patient, we often don’t even have to touch a patient, physically or emotionally. It may interfere with collection of psychosocial and emotional information and, therefore, with development of supportive, healing relationships. On the other hand, patient access to the electronic health record and messaging functions may improve communication, patient empowerment, engagement and self-management.

Redesigning patient experience measurement and assessing our organisations’ readiness to deliver on the promise of patient centredness will empower the healthcare professionals to deliver not just care, but actual caring. There was substantial evidence that physicians’ beliefs and attitudes were demonstrated as significant physician-related factors influencing patient participation in medical decisions. Although researchers used different approaches to implement PCC interventions, there has been no comparison of patient-centredness and its outcomes in different European countries. Therefore, we need good large databases and interventional studies on population level.

# Abstract: Person-centred care, a core concept in family medicine

**Authors:** Igor Švab

**Affiliations:** Medical Faculty, University of Ljubljana, Slovenia

## Abstract

Person-centred care is a crucial concept that was first defined within the scope of defining family medicine. It is considered one of the main characteristics of family medicine. Its definition was primarily established by EURACT. By defining the concept, it could be taught and incorporated into medical education curricula.

However, the concept of person-centred care is only meaningful if evidence is produced to prove its usefulness in healthcare. This is the task of research organisations, which need to provide evidence that person-centred care works and positively impacts healthcare quality. Even when this evidence is provided, convincing policymakers and other decision-makers to implement person-centred care in healthcare policies remains a significant challenge.

The future of patient-centred care will likely to involve even greater emphasis on personalisation, technology and patient engagement. By leveraging these tools to deliver more individualised and responsive care, healthcare providers can improve health outcomes and patient satisfaction while also reducing healthcare costs.

# Abstract: Family medicine as a scientific and professional discipline in Croatia

**Authors:** Venija Cerovečki

**Affiliations:** Department of Family Medicine, School of Medicine, University of Zagreb; Zagreb-Centar Health Center

## Abstract

Croatian family medicine has a rich and challenging past but also a challenging present and future. Analysing the development of family medicine in Croatia, it can be concluded that the profession has always followed scientific achievements, implemented them in theoretical training and scientifically analysed them but has encountered and encounters difficulties in implementing scientific findings in everyday practice. This challenge requires a lot of effort in the future related to convincing decision-makers and stakeholders that scientific achievements and their implementation in daily practice improve quality of care for the whole population.

Family medicine as a scientific discipline was primarily developed within the activities of the Departments of Family Medicine, first within the Department of Family Medicine in Zagreb and then at the Departments of Family Medicine in Split, Rijeka and Osijek. These activities implied involvement of members of the Departments of Family Medicine in the preparation of dissertations but also in the implementation of international and national research projects, which were often connected with membership in European General Practice Research Network (EGPRN). Scientific projects are carried out through the activities of members of the Departments of Family Medicine as well as other family doctors in clinical and public health projects, which also promotes an interdisciplinary approach in family medicine in scientific activities. Family medicine as a professional discipline was formally developed per international recommendations.

The fact that speciality training as a necessary component of the profession’s development was conceptually conceived at School of Public Health ‘Andrija Štampar,’ School of Medicine, University of Zagreb in 1960, confirms Croatian family physicians as active participants in the development of the profession of family medicine on a global level. Since then, the speciality training of family physicians has been carried out and improved based on the recommendations of European Academy of Teachers in General Practice/Family Medicine (EURACT), European Union of General Practitioners/Family Physicians) UEMO and other international associations of medical education, primarily An International Association of Health Professional Education (AMEE).

# Abstract: Photovoice-inspired qualitative analysis of photographs from family physicians’ practice in Italy: A pilot study

**Authors:** Alice Serafini, Irene Bruschi, Giuseppe Parisi, Viviana Forte, Luca Lenzotti, Mirko Pasquini, Martina Belluto, Luca Ghirotto

**Affiliations:** University of Modena and Reggio Emilia, Modena, Italy

**Keywords:** Healthcare architecture, humanisation, photovoice

## Abstract

**Background:** The environment and furniture of a medical workplace can influence both patient and health team satisfaction and healthcare structures projected carefully are an important point for humanisation of care. Several studies have been performed in hospital settings but only a few in primary care.

**Research question**: How do family physicians choose to equip their practice? What do they think about their practice equipment and furniture?

**Methods**: We designed a qualitative pilot study inspired by the photovoice methodology. We collected data through an anonymous online survey using a snowball sampling approach. In the first part of the survey, we collected socio-demographic variables. In the second part, we asked participants to upload: first, one or two pictures of their practices and explain what they liked or not and what they would have changed, second, a picture of the most important thing in their opinion in the practice adding the explanation why they made that choice. Written answers were analysed using thematic analysis, while participants’ characteristics through descriptive statistics. A pilot analysis of the visual material is ongoing.

**Results**: In the survey, 140 GPs participated but we obtained only 30 complete responses (79.17% dropout rate). With the thematic analysis of the written responses, we identified five main themes: environment and atmosphere, customisation, furniture and objects, collaboration, hygiene. Each theme contains sub themes that describe positive and negative aspects.

**Conclusion**: The participants’ answers suggested helpful tips for setting up a GP practice. It is important to create a welcoming environment, being careful in choosing colours and furniture and not forgetting to add plants. The practice should include a space for a bedvisit and a proper privacy and a space for consultation. Finally, a customised environment, including personal objects, resulted central for GP motivation.

# Abstract: Person-centred care for patients with long COVID

**Authors:** Jako Burgers, Jeroen Gruiskens, Thijs Van Meulenbroek, Annerika Gidding-Slok, Ivan Huijnen, Cynthia Lamper, Jean Muris, Marcia Spoelder-Merkens, Darcy Ummels, Carlijn Wiertz, Thomas Hoogeboom, Jeanine Verbunt

**Affiliations:** Department Family Medicine, Maastricht University, Maastricht, Netherlands

**Keywords:** Person-centred care, long COVID, rehabilitation

## Abstract

**Background:** More than 8 million people in the Netherlands have experienced COVID-19. Approximately 200,000 people have had persistent symptoms for over four weeks (long COVID). Depending on the complexity of problems and limitations in daily life, the patient is treated by the general practitioner (GP), supplemented with allied healthcare (e.g. physiotherapy, occupational therapy, speech therapy) or specialised rehabilitation care. The effectiveness of these treatments, however, is unknown.

**Research question:** Is a person centred care programme useful in improving societal participation? How should a regional interdisciplinary COVID-19 rehabilitation network be designed?

**Methods:** First, a pragmatic literature review of studies and guidelines on long COVID was performed. Then, we organised five focus groups with patients and four focus groups with healthcare professionals to explore their views and experiences of current healthcare. Both review outcomes and focus group findings will be used in designing care pathways specified by level of complexity. These pathways will be evaluated in single-case experimental design studies (SCEDs).

**Results:** The project runs from August 2022 to February 2024. The literature review yielded 53 studies and three guidelines (Dutch, NICE, WHO). No studies with strong evidence of quality were found. Research on specific rehabilitation programs is lacking. Most guideline recommendations were based on low evidence or expert opinion. In general practice, lifestyle advice, support in return to work and monitoring are recommended. Patients in focus groups indicated that GPs have little knowledge of long COVID. Most rehabilitation treatments included energy management techniques (i.e. pacing). Patients were satisfied with allied healthcare professionals. Patients and professionals agreed that collaboration and patient involvement in managing care is essential in complex cases.

**Conclusion:** Long COVID care needs improvement of knowledge and specific recommendations for GPs. Core values (e.g. person-centredness, continuity and a holistic approach) seem essential in effectively supporting patients.

# Abstract: Maltreatment of older people: Challenges for patient-centred care in general practice

**Authors:** Lieve Peremans, Naomi Aerts, Kristof Van Assche, Maxime Castermans, Paul Van Royen, Hilde Bastiaens

**Affiliations:** Primary and Interdisciplinary Care, University of Antwerp, Antwerp-Wilrijk, Belgium

**Keywords:** Maltreatment, older people, patient-centred care

## Abstract

**Background:** Older people are vulnerable to maltreatment but also reluctant to ask healthcare providers for support. Interprofessional collaboration between healthcare and justice has deontological obstacles. Limited research has been done on models considering the patient’s perspective.

**Research question:** What are the different stakeholder’s perspectives and concrete obstacles in the prevention, detection and policy for maltreatment?

**Methods:** The first step involves a descriptive qualitative interview study with a purposive sample of patients and experienced professionals, including nurses, GPs, social workers, notaries, lawyers and judges. Two skilled interviewers (NA and MC) started data collection in October 2022. Respondents were identified by organisations familiar with the problem and by snowballing. Second, we are starting a multiple case study of abused older persons to identify and interview the parties involved and examine the obstacles they encountered during the intervention process.

**Results:** Twenty-four patients and 39 professionals were interviewed. Three main themes were highlighted: (1) need for sensitisation of older people and professionals, (2) difficulties raised by deontological obligation and (3) need for and obstacles hampering interprofessional collaboration.

Multi-level sensitisation is needed because of the complexity of elder abuse; the victim’s vulnerability, reluctance to request help; and potential abuse of powers of attorney.

Healthcare professionals focused on a patient-centred approach, identified a tension between a trust-based relationship and upholding professional secrecy. There is no adequate model for interprofessional collaboration but only the individual actors’ goodwill.

**Conclusion:** These results highlight the complexity of elder abuse situations and the challenges in developing and valorising an interprofessional collaboration model which can overcome the obstacles for collaboration between healthcare, welfare and legal experts and which takes into account the patient’s perspective.

# Abstract: Patient-centred interprofessional collaboration and integration in primary care. A qualitative study

**Authors:** Muhammed Mustafa Sirimsi, Kris Van Den Broeck, Hans De Loof, Kristel De Vliegher, Peter Van

**Affiliations:** Bogaert University of Antwerp, Merksem, Belgium

**Keywords:** Integrated care, interprofessional collaboration, patient-centred care, health economics, primary care, public health

## Abstract

**Background:** Interprofessional collaboration and integration (IPC&I) is a crucial aspect of primary care (PC), where patients often require the coordinated efforts of multiple caregivers. To achieve this, effective and patient-centred communication (PCC) is necessary to ensure that the patient’s needs and preferences are central to the care process. PCC has been shown to enhance patient satisfaction and improve health outcomes.

**Research question:** How do caregivers experience the treatment of chronic patients regarding patient-centred IPC&I in PC? What are the needs and preferences of caregivers to improve IPC&I in PC? How to establish a patient-centred IPC&I in PC?

**Methods:** This study used a qualitative descriptive design with thematic analysis and explored caregivers’ experiences regarding IPC&I in PC. Five focus groups (FGs) were performed in three waves, with 36 caregivers, academics, policymakers and patient organisations, using maximum variation sampling. Wave one included an FG with five participants (academics and/or caregivers), wave two consisted of three separate FGs, with 23 participants (academics, patient organisations, and policymakers), and wave three was performed with seven participants chosen from the second wave.

**Results:** The participants represented a broad range of disciplines working in PC, ensuring a multidisciplinary character of the FGs. The interviews lasted between 68 and 123 min and the research resulted in the following five themes: (i) Readiness and attitude towards IPC&I, (ii) IPC&I to improve the quality of care, (iii) having a patient-centred IPC&I, (iv) the need for education to normalise IPC&I, and (v) financial systems – practice organisation.

**Conclusion:** Overall, the study provides valuable insights into the caregivers’ experiences in treating chronic patients in PC through patient-centred IPC&I and the need for improvement in this area. The findings of this study can inform the development of policies and education programmes to improve the quality of care for chronic patients in PC.

# Abstract: Perceptions of illness in patients diagnosed with type 2 diabetes mellitus

**Authors:** Lucija Gosak, Mateja Lorber, Gregor Stiglic

**Affiliations:** Faculty of Health Sciences, University of Maribor, Maribor, Slovenia

**Keywords:** Type 2 diabetes mellitus, disease perception, person-centred care

## Abstract

**Background:** The perceptions and beliefs of individuals with type 2 diabetes mellitus and their knowledge influence is important for individual’s health. The perception of the illness is also associated with the quality of life and the state of the illness. Patients with chronic conditions require person-centred care to meet the patient’s health needs and achieve a better quality of life.

**Research question:** What is the perception of the illness in patients diagnosed with type 2 diabetes mellitus?

**Methods:** A questionnaire Brief Illness Perception Questionnaire (Broadbent et al. 2006) was used, which provides a rapid assessment of disease perception. The scale measures a patient’s cognitive and emotional representations of their illness, including consequences, timeline, personal control, treatment control, identity, compliance, care, emotional response and causes. We performed a categorical analysis for the highest stated cause of the disease.

**Results:** In the study, 141 people diagnosed with T2DM participated. The mean age of the participants was 63.08 (SD =12.96). The mean perception of T2DM score was 40.06/80 (SD =10.46), which does not reflect a very threatening view of the disease. A more threatening perception of the disease was held by participants who, in addition to diabetes, also reported obesity (41.08 vs. 40.78) and the presence of cardiovascular disease (42.10 vs. 41.15). The most important factors influencing their disease were heredity and genetics (*n* = 33), stress and other psychological strains (*n* = 24) and inadequate diet (*n* = 23).

**Conclusion:** Most of the respondents feel that they can influence their disease themselves, so they must receive ongoing care and support from a nurse to change their behaviour and a person-centred individual approach. Most person-centred methods are developed targeting personal consultations, although group-based programmes are a widespread and efficient method of support. Person-centredness in group-based programs requires a change in practice towards addressing biopsychosocial issues and facilitating group processes, which can lead to a better perception of chronic disease.

# Abstract: An e-learning course on the patient-centred approach to colorectal cancer screening: GP’s first choice!

**Authors:** Amélie Aïm-Eusébi, Antoinette Bouziane, Karima Sekri, Bernard Clary, Bernard Freche, Julien Le Breton, Isabelle Auger

**Affiliations:** Université Paris Cité, Paris, France

**Keywords:** Person-centred care, colorectal cancer, screening, e learning, primary care

## Abstract

**Background:** Colorectal cancer (CRC) is the third most common cancer in men and the second in women worldwide. Between 2020 and 2021, in France, 35% of the eligible population completed screening, which is under the acceptable uptake rate.

**Research question:** The FORCEPS study aimed to test whether a training programme for general practitioners (GPs) focused on a person-centred approach could increase their patients’ CRC screening rate. Our study reports on the construction of, participation in and evaluation of the e-learning course tested in the FORCEPS study.

**Methods:** An interdisciplinary team designed an e-learning programme for French GPs on the Moodle® platform. Learning activities related to the stated learning objectives and interactive approaches were specified. Two sessions took place, in May and November 2018, for six weeks. We evaluate the intended knowledge and skills acquired by the participants and their satisfaction. We used an online mixed-method survey with Likert scales and open-ended questions.

**Results:** In total, 116 GPs registered for the training programme, 70% of the GPs who registered for the first session opted to follow the training *via* e-learning rather than in person. Among the participants, 36.0% followed the training in its entirety during session 1, as did 24.0% during session 2. At the end of the training, none of the participating GPs self-assessed themselves as a ‘novice,’ and the number of GPs self-assessing as ‘intermediate’ or ‘experienced’ increased. 62.9% of the participants were generally satisfied with the e-learning course.

**Conclusion:** The web-based programme focused on improving GPs’ communication skills to encourage CRC screening was chosen by most participating GPs and received moderately positive reviews. While GPs’ theoretical knowledge can be improved through our training programme, it remains to be seen whether or not this will be reflected in their everyday practice.

# Abstract: Health professionals’ role in providing patient-centred and equitable care to LGBTIQ + patients: Translation, cultural adaptation and validation of the Greek version of Lesbian, Gay, bisexual and transgender development of clinical skills scale

**Authors:** Ilias Pagkozidis, Bettina Haidich, Loukas Athanasiadis, Theodoros Dardavesis, Zoi Tsimtsiou

**Affiliations:** MSc Social– Preventive Medicine and Quality in Healthcare, Laboratory of Hygiene, Social & Preventive Medicine and Medical Statistics, School of Medicine, Aristotle University of Thessaloniki, Greece, Thessaloniki, Greece

**Keywords:** Quality in healthcare, patient-centredness, equity, validation, LGBTIQ+, LGBT-DOCSS

## Abstract

**Background:** Ensuring patient-centred and equitable care is intertwined with quality in primary care and important for minorities, such as the LGBTIQ + community, often described as having limited access, lower satisfaction with health services, and worse health outcomes. This study aimed to provide a valid and reliable Greek version of a tool, allowing the recording of health professionals’ knowledge, attitudes and clinical preparedness towards LGBTIQ + patients.

**Research question:** Is the Greek version of the Lesbian, Gay, Bisexual, and Transgender Development of Clinical Skills Scale (LGBT-DOCSS) valid and reliable?

**Methods:** After obtaining permission by the scale’s developer, bilingual translation was performed, followed by cultural adaptation, which shaped the Greek version of LGBT-DOCSS, after interviewing 13 health professionals. A validation study was carried out among health professionals at Hippocratio General Hospital of Athens. The validity of the Greek version was tested with structural validity (convergent and known groups validity) and face validity while internal consistency and test-retest reliability (second completion after 2–3 weeks by same participants) were applied to test reliability.

**Results:** Overall, 238 healthcare professionals participated in the study. The Greek LGBT-DOCSS demonstrated known group validity, with groups characterised by older age, stronger religiosity, no contact with LGBTIQ + individuals at work or personal life, no LGBTIQ + health education, and heterosexual orientation being associated with lower scores (*p* < 0.05). Convergent validity was demonstrated (correlation with the Greek version of Attitudes towards Lesbians and Gay men (ATLG) scale, Spearman’s rho = –0.598, *p* < 0.001) while face validity was confirmed by 81.9% (*n* = 195) of participants (median 8/10). Regarding reliability, satisfactory internal consistency (Cronbach *a* = 0.785) and test-retest reliability (Pearson’s *r* = 0.793 and student’s *t*-test *p* = 0.7) were found.

**Conclusion:** The Greek version of LGBT-DOCSS is a valid and reliable scale, the only tool available in Greece for mapping health professionals’ knowledge, readiness and attitudes towards LGBT patients to ensure the provision of patient-centred and equitable care.

# Abstract: Health-related quality of life during the pandemic in Germany: A web-based longitudinal study

**Authors:** Dominik Schröder, Stephanie Heinemann, Gloria Heesen, Alexandra Dopfer-Jablonka, Frank Klawonn, Frank Mueller

**Affiliations:** University Medical Center Göttingen, Göttingen, Germany

**Keywords:** COVID-19, long COVID, pandemic, Quality of Life, social participation

## Abstract

**Background:** Health-related Quality of Life (hrQoL) is important when characterising and comparing health over time. For new diseases as long COVID, hrQoL can give insights into terms of primary care demand and treatment, especially when investigated in a longitudinal study design.

**Research question:** How does hrQoL changes throughout the pandemic in individuals with lasting long COVID compared to participants without previous SARS-CoV-2 infection? How do symptoms change in participants with long COVID?

**Methods:** An online survey was conducted in Germany investigating changes in hrQoL and symptoms using a longitudinal study design including two-time points. All persons 18 years or older were eligible to participate. EQ-5D-3L and EQ-VAS were used as hrQoL outcome. 27 symptoms were rated by participants with long COVID on a 11-point Likert-scale (0 = no symptoms; 11 = worst symptoms). Covariates include sociodemographic, medical and pandemic-specific variables. Descriptive and inferential statistics were performed comparing changes in hrQoL between both time points and across groups according to their COVID-19 status.

**Results:** Data from 1,113 participants was included in the analysis. The average time between baseline and follow-up was approximately 60 days. Between baseline and follow-up participants with Long COVID (*n* = 699) worsened significantly in EQ-5D VAS scores (M = −2.14, SD =18.13, *p* < 0.001) and improved significantly in EQ-5D-3L index scores (*M* = 0.04, SD =0.18, *p* < 0.001). In participants with no previous SARS-CoV-2 infection (*n* = 329) hrQoL did not change significantly. The health dimensions pain/discomfort and anxiety/depression improved the most in participants with long COVID, 20 out of 27 symptoms in participants with long COVID improved significantly regarding symptom severity and prevalence.

**Conclusion:** Subjective hrQoL worsened while objective hrQoL improved in participants with long COVID. This discrepancy needs to further investigation as subjective hrQoL determines primary care demand. EQ-5D index score may not reflect all health dimensions related to long COVID.

# Abstract: Demographic and behavioural characteristics of populations vaccinated against COVID-19 with and without prior influenza vaccines, aged 65 and above: A combined cross-sectional and survey study

**Authors:** Galia Zacay, Anthony Heymann, Danna Valinsky

**Affiliations:** Meuhedet HMO, Ganei-Tikva, Israel

**Keywords:** Immunisation, patient adherence, public health, community medicine, doctor-patient relationship

## Abstract

**Background:** The proportion of the population vaccinated in Israel against COVID-19 during 2021 was extremely high compared with the influenza vaccine uptake rates. Understanding the precipitating factors that led to the high vaccination rate may facilitate maintaining these rates in the event of a recommendation for a seasonal COVID-19 vaccine.

**Research question:** This study aimed to identify factors associated with repeat influenza vaccination that may assist in maintaining high adherence to the COVID-19 vaccine in the future.

**Methods:** We used a mixed-methods design for this study. The first part was a cross-sectional study of adults aged 65 and over vaccinated against COVID-19, comparing variables such as age, gender and health status between those with a history of influenza vaccination in the previous five years and those without. The second part consisted of a questionnaire administered to a subsample of the above population regarding vaccine hesitancy and intention to be vaccinated.

**Results:** Strong adherence to annual influenza vaccine recommendations was associated with earlier COVID-19 vaccine uptake. Regardless of previous influenza vaccine history, all groups had a high uptake of the third COVID-19 booster vaccine. Respondents with lower adherence to influenza vaccines were more likely to demonstrate higher levels of vaccine hesitancy but despite this, motivators for the COVID-19 vaccine were similar between groups.

**Conclusion:** Confidence in the health system and the COVID-19 vaccine is a strong motivator regarding vaccine uptake. This should be addressed to maintain high vaccination rates.

# Abstract: Eurodata study: COVID-19 vaccination pathway and the role of primary care in 27 European countries

**Authors:** Lourdes Ramos Del Río, Ileana Gefaell, Sara Ares Blanco, Marina Guisado Clavero, Louise Fitzgerald, Heidrun Lingner, Liubovė Murauskienė, Ábel Perjés, Davorina Petek, Ferdinando Petrazzuoli, Goranka Petricek, Martin Sattler, Natalija Saurek-Aleksandrovska, Alice Serafini, Theresa Sentker, Gunta Ticmane, Peter Torzsa, Bert Vaes, Shlomo Vinker, Limor Adler, Maria Bakola, Sabine Bayen, Elena Brutskaya-Stempkovskaya, Iliana Carmen Busneag, Asja Ćosić Divjak, Maryher Delphin Peña, Philippe-Richard Domeyer, Sabine Feldmane, Dragan Gjorgjievski, Miroslav Hanževački, Kathryn Hoffmann, Oksana Ilkov, Shushman Ivanna, Marijana Jandric-Kocic, Erva Ucuncu, Aleksandar Kirkovski, Snežana Knežević, Büşra Çimen Korkmaz, Milena Kostic, Anna Krztoń-Królewiecka, Liga Kozlovska, Katarzyna Nessler, Anna Segernäs Kvitting, Sven Streit, Senn Oliver, Raquel Gomez Bravo, Maria Pilar Astier-Peña, Ana Luisa Neves, Nagu Penakacherla, Bruno Heleno, Sherihane Bensemmane

**Affiliations:** SERMAS, Madrid, Spain

**Keywords:** COVID-19, primary care, vaccination, drug-related side effects and adverse reactions

## Abstract

**Background:** Primary health care (PHC) participated in COVID-19 vaccination in Europe but their role has not been described.

**Research question:** How was the COVID-19 vaccination pathway in different European countries in PHC? Where did vaccination take place in the community?

**Methods:** Descriptive, cross-sectional, retrospective study with qualitative data acquired through a semi-structured questionnaire to know COVID-19 vaccination in PHC in Europe (27 countries participating). Main variable: COVID-19 vaccination pathway in PHC. All variables were collected from each country from December 2020 to December 2021.

**Results:** Vaccination centres were developed in 25 countries. PHC professionals participated in the vaccination campaign in 25 countries but the participation was variable (in Italy was voluntary, in Turkey and Greece, only adults were vaccinated in PHC). GP practices vaccinated in 20 countries. GPs and nurses were more frequently involved but also midwifes, pharmacists, paediatricians, dentists, physiotherapists, students, paramedics. Patients could get an appointment online, by phone or in person in most countries. A safety protocol before vaccination was present in nearly all countries. Advice regarding side effects was given in all nations with informed consent in at least three countries. The waiting time after the vaccination was between 10 and 30 min. Patients with prior anaphylactic reactions were referred to Allergology in six countries. Nurses and doctors provided care in case of vaccine side effects.

**Conclusion:** Multiprofessional PHC teams participated actively in the COVID-19 vaccination in at least 25 European countries.

# Abstract: Psychological determinants of vaccination behaviour against COVID-19 and influenza of chronically ill in German primary care – A cross-sectional survey

**Authors:** Linda Sanftenberg, Simon Keppeler, Nadine Heithorst, Tobias Dreischulte, Marco Roos, Philipp Sckopke, Markus Bühner, Jochen Gensichen

**Affiliations:** Institute of General Practice and Family Medicine, Munich, Germany

**Keywords:** Primay care, mental health, chronically ill, vaccination, behaviour change

## Abstract

**Background:** Vaccines against COVID-19 and influenza are safe and provide good protection from severe infections. They are highly recommended for vulnerable patients with chronic diseases. These patients often suffer from psychological comorbidities like depression and anxiety disorders. Chronically ill adults are mainly vaccinated in primary care, but vaccination rates remain insufficient.

**Research question:** This study aims to analyse the associations of depression and anxiety disorder on vaccination behaviour against COVID-19 and seasonal Influenza in chronically ill primary care patients.

**Methods:** For this cross-sectional observational study, a paper-based survey was conducted from July- December 2022 in Bavaria, Germany. We invited adult primary care patients suffering from one or more chronic health conditions (type 1/2 diabetes, asthma bronchial/chronic obstructive pulmonary disease, coronary heart disease or breast cancer). Besides sociodemographics, we analysed symptoms of depression (PHQ-9) and anxiety disorder (OASIS), social activity (LSNSN), patient activation (PAM), physician-patient relationship (PRA) and antecedents of vaccination behaviour (5 C). Descriptive statistics and linear mixed-effects regression models were calculated.

**Results:** We analysed data of 795 study participants. Major depression was suspected in 18.4%, and anxiety disorder in 20.3%. Concerning vaccinations against COVID-19, depression was negatively associated with trust in vaccine safety (item ‘Confidence’ *β* = −0.04, 95% CI [–0.07, −0.01]) and positively associated with subjectively perceived structural barriers to getting vaccinated (item ‘Constraints,’ *β* = 0.02, 95% CI [0.001; 0.04]). Concerning vaccinations against influenza, depression did not show any association with vaccination behaviour. Higher age, male sex, higher education, high scores of self-activation, and an excellent physician-patient relationship were associated with positive vaccination behaviour.

**Conclusion:** To address low confidence in the safety and efficacy of vaccinations, targeted educational interventions like communication-based short interventions in primary care might be helpful. Subjectively perceived constraints might be addressed by regular personalised reminder systems.

# Abstract: How the COVID-19 pandemic affected the total annual time spent by family physicians managing common diagnoses

**Authors:** Shlomo Vinker, Avivit Golan Cohen, Ilan Green, Eugene Merzon, Ariel Yehuda Israel

**Affiliations:** Family Medicine, Leumit Health Services, Ashdod, Israel

**Keywords:** Workload, reason for encounter, complaint, COVID-19

## Abstract

**Background:** The COVID-19 pandemic changed how common diseases are diagnosed and managed. Telemedicine modalities have been increasingly used. COVID-19 may have triggered new complaints, while lockdowns, social isolation, and barrier precautions adopted during the pandemic may have decreased the transmission of several common infectious diseases.

**Research question:** To study changes in the total annual time spent by primary care physicians between 2019 and 2022 by dividing visits according to treated complaints categories.

**Methods:** A cross-sectional study based on the electronic medical records of all patients of LHS visits to primary care physicians in 2019 and 2022 (*n* = 715,000 patients).

Visits had been classified according to the ICD-9 code of the visit. For each code, we calculated AADT (Accumulated Annual Duration of Time), and we calculated the change of AADT in each ICD-9 code/codes group between 2019 and 2022.

**Results:** We observed a marked increase in the AADT spent for visits for poorly defined complaints and administrative tasks (+26%). There was also a substantial increase in AADT spent in visits for metabolic complaints such as obesity (+24%), diabetes mellitus (+6.1%), and gastrointestinal problems (+2.9%). Interestingly, less AADT was spent on respiratory infections (–30%), COPD (–23%), ophthalmological problems (–11.6%), and ear problems (–4.6%). Also less AADT was spent in visits related to injuries (–14%), arthropathies (–4.5%), and back problems (–2.1%). Less AADT was also recorded for treating chronic conditions such as hypertension (–21%), disease screening and health promotion encounters (–5.3%).

**Conclusion:** The reduction of respiratory infectious diseases is temporary but the shift from face-to-face medicine may have led to a concerning trend of spending more time on administrative tasks and shifting the treatment of ENT and Ophthalmologic problems to other specialists. These trends result from external forces; it is time to be proactive in influencing the case mix in our clinics.

# Abstract: Social participation and mental health of immunocompromised individuals during the COVID-19 pandemic – Results of a longitudinal observational study over three-time points

**Authors:** Gloria Heesen

**Affiliations:** Institute for General Practice, Göttingen, Germany

**Keywords:** Immunocompromised persons, social participation, mental health, quality of life, observational study, SARS-CoV-2 vaccination, pre/post design

## Abstract

**Background:** The pandemic of the coronavirus disease 2019 (COVID-19) impacted how people perform their daily lives in manifold and sometimes massive ways. Notably, individuals at high risk for severe disease progression, like immunocompromised people, may have experienced drastic changes in social participation during the pandemic. A basic COVID-19 vaccination may have changed their safety behaviour in terms of infection risk and thereby influence social participation and mental well-being.

**Research question:** How does the self-perceived social participation change in immunocompromised individuals at baseline before and at follow-up one and six months after basic vaccination?

**Methods:** Beginning in March 2021, 274 immunocompromised persons 18 or older were enrolled in the COVID-19 Contact Immune study in Lower Saxony, Germany. Measurements were performed at three-time points regarding social participation (Index for the Assessment of Health Impairments [IMET]), mental health (Patient Health Questionnaire-4 [PHQ-4]), subjective health status (five-point Likert-scale) and quality of life (five-point Likert-scale).

**Results:** About 60% of the participants showed increasing social participation over time. The greatest increase in social participation was observed within the first month after basic vaccination (*p* < 0.001). During the following five months, social participation remained stable. The domains ‘Social activities,’ ‘Recreation and leisure’ and ‘Close personal relationships’ were responsible for the overall change in social participation. No association was found between social participation, mental health, socio-demographic or medical factors (except hypertension).

**Conclusion:** The protection expected of a COVID-19 vaccination is likely to have increased social participation. These results highlight the heterogeneity of changes in social participation during a similar period. It is helpful for general practitioners to address health-related consequences of more social participation among immunocompromised persons. Further factors that might influence social participation need to be investigated to be able to advise and treat patients in standard care better and more individually.

# Abstract: A COVID-19 Conundrum – Can the reported skin manifestations of COVID-19 to be explained by re-activation of herpes virus?

**Authors:** Itamar Getzler

**Affiliations:** Maccabi Health Services, Kfar Saba, Israel

**Keywords:** COVID-19, herpes, herpesviridae, SARS-COV-2

## Abstract

**Background:** Since COVID-19 outbreak, numerous reports concerning COVID-19 skin manifestations have emerged. A debate has ensued, lacking evidence to support whether these skin manifestations are unique or represent a form of re-activation with Herpesviridae viruses.

**Research question:** The goal of this research was to compare the incidence of herpetic skin lesions between COVID-19 group, Vaccinated group, and their respective control, considering Herpesviridae carrier status.

**Methods:** Maccabi Health Services’ database was digitally scanned and a cohort of both verified COVID-19 and SARS-COV-2 vaccinated patients were extracted in respect to the study timeframe. Random control cohorts were pulled from the database to be matched to the cohorts. All patient records of the cohort were then analysed to find occurrences of clinically diagnosed herpetic lesions during and before the study timeframe.

**Results:** For each cohort, 105,000 patients were recruited. For the COVID-19 cohort, we demonstrated a non-significant difference between both groups for developing herpetic skin lesions. A logistic regression showed that the Interaction of COVID-19 with History of Herpes is statistically significant with an odds ratio of 0.51 (*p* = <0.001). For the ‘Vaccination’ cohort, the data shows statistically significant differences between groups in herpetic frequencies but contrary to the COVID-19 disease, the interaction of the vaccine with the ‘history of herpes’ is not significant with an odds ratio of 0.74 (*p* = <0.070).

**Conclusion:** We can infer that COVID-19 has a low or non-significant impact on the development of clinically diagnosed herpetic lesions within one month of diagnosis. This result diminishes the idea of COVID-19 contributing to unique skin lesions. However, because of the meaningful interaction of the COVID-19 disease with the History of herpes variable, we can suggest that COVID-19 has a role in re-activation of latent Herpesviridae infections. The SARS-COV-2 vaccine can increase the odds of developing herpetic infection almost 2-fold relative to those who did not get the vaccine.

# Abstract: Changes in work tasks and organisation of general practice in Norway during the COVID-19 pandemic: Results from the Pricov-19 study

**Authors:** Torunn Bjerve Eide, Esther Van Poel, Sara Willems, Frode Fadnes Jacobsen

**Affiliations:** Department of General Practice, Institute of Health and Society, University of Oslo, Oslo, Norway

**Keywords:** COVID-19, PRICOV-19, quality of care, infection prevention and control, patient flow management, primary care workforce

## Abstract

**Background:** The COVID-19 pandemic led to huge and rapid changes in general practice in Norway as in the rest of Europe. Knowledge regarding these changes can inform measures in similar future situations.

**Research question:** We aimed to obtain more knowledge on the changes in use of alternative consultation forms, workload, tasks and delegated responsibilities of both the GPs and other personnel in the GP offices, adaptations in routines related to hygiene measures, triage of patients, and how the official rules and recommendations affected the practices.

**Methods:** We analysed data from the Norwegian part of the PRICOV-19 study, collecting data from general practice *via* an online self-reported questionnaire. We included 130 Norwegian general practices, representing an estimated 520 Norwegian general practitioners (GPs). All Norwegian GPs were invited to participate.

**Results:** During the pandemic, Norwegian GPs significantly increased their use of alternative consultation forms and implemented infection prevention measures in their clinics. There was a large and significant increase in the use of all alternative consultation forms (digital text-based, video- and telephone consultations). The use of several different infection prevention measures was significantly increased, and the provision of hand sanitiser to patients increased from 29.6% pre-pandemic to 95.1% since the pandemic. More than half of the GPs (59.5%) reported that their responsibilities in the practice had increased, and 41% were happy with the task shift. 27% felt that they received adequate support from the government; however, 20% reported that guidelines from the government posed a threat to the well-being of the practice staff. We found no associations with the rurality of the practice location or size of the municipalities.

**Conclusion:** Norwegian GPs adapted well to the need for increased use of alternative consultation forms and reported a high acceptance of their increased responsibilities – however, only one in four experienced adequate support from the government.

# Abstract: Innovative population-based strategies for primary prevention of cardiovascular disease: A 2-year randomised control trial (RCT) evaluating behavioural change led by community cham-pions versus brief advice

**Authors:** Delphine Le Goff, Gabriel Perraud, Jérémy Derriennic, Paul Aujoulat, Morgane Guillou, Marie Barais, Jean Yves Le Reste

**Affiliations:** Faculté de médecine, Médecine Générale, Brest, France

**Keywords:** Primary prevention, cardiovascular system, community participation, community health workers, motivational interviewing

## Abstract

**Background:** Cardiovascular diseases (CVD) cause 17.9 million deaths worldwide. The SPICES project implemented a cardiovascular disease prevention community-based program. During the summer of 2019, students screened the population with the Interheart Non-Laboratory Score (INL) to recruit participants in a French rural and vulnerable setting. Then, community champions were trained in behavioural change. This study aimed to assess their efficacy with intermediate INL adults.

**Research question:** The primary objective of this study was to evaluate the efficacy of a behavioural change programme plus brief advice conducted by community champions, for people at intermediate cardiovascular risk, compared with brief advice only.

**Methods:** A randomised control trial with 1:1 allocation tested brief advice plus behavioural change led by community champions versus brief advice. A 24-month difference of 15% in the INL in intention to treat was expected. Participants’ objectives were independently recorded at four months.

**Results:** In total, 1,309 participants met the inclusion criteria, 536 people were analysed. In March 2021, the COVID-19 pandemic happened with three lockdowns during the trial and public restrictions. At 24 months, 110 people remained in intervention group and 147 in control group (total 257). Differences within groups were not significant (–0.12 (–0.80; 1.04) *p* = 0.758). The main participants’ objective was weight-loss. Following the hierarchical analysis, the confirmatory analysis procedure could not continue beyond the first non-significant criterion. On an exploratory basis, no significant difference was found in the secondary criteria. Only 29% of eligible subjects agreed to participate in the study. Among those, only 48% completed the study.

**Conclusion:** The difference in the mean INL score of 15% was not achieved. Money for health procedures would be better invested in public procedures. People entering individual strategies should be sorted on their capacities to change. The investment to accompany participants did not lead to success despite the sociological literature evidence.

# Abstract: The treatment results of hypercholesterolaemia and costs of care for prediabetics compared with type 2 diabetics in Southwest Finland

**Authors:** Merja Laine, Hannu Järveläinen, Markku Vielma, Juha-Matti Varjonen, Päivi Rautava

**Affiliations:** University of Turku, Paimio, Finland

**Keywords:** Prediabetes, diabetes, comorbidities, treatment, costs

## Abstract

**Background:** Prediabetics have a markedly increased risk of developing type 2 diabetes and cardiovascular diseases. Moreover, the treatment costs caused by type 2 diabetes and its comorbidities are high.

**Research question:** Does the intensity of lipid treatment differ in prediabetics and type 2 diabetics if cardiovascular diseases were established/not diagnosed? How do macrovascular complications affect the costs of treatment?

**Methods:** This was a qualitative retrospective register study. The study included data from 37,501 type 2 diabetics and 42,554 prediabetics from Southwest Finland in 2019. Prediabetics were identified based on abnormal glucose metabolism from the patient registers of the health centres. Diabetics were identified and grouped based on laboratory tests, diagnosis and special reimbursement rights for antidiabetic drugs. Cardiovascular diseases were identified based on the ICD10 diagnosis from the historical data. Treatment levels for LDL cholesterol and costs were compared in type 2 diabetics and prediabetics with and without cardiovascular comorbidities.

**Results:** In total, 32% (11,998) of type 2 diabetics and 26% (10,892) of prediabetics had cardiovascular comorbidities. LDL was studied in 77% of type 2 diabetics and 66% of prediabetics. Only 40% of diabetics and 30% of prediabetics with cardiovascular disease, achieved a target of LDL <1.8 mmol/l. More than half of those without complications had LDL >2.5 mmol/l. The total costs of specialised medical care and pharmacotherapy per patient per year for type 2 diabetics with and without complications were 4,688 euros and 1,981 euros, respectively; and for prediabetics 3,376 and 1,961 euros, respectively.

**Conclusion:** Treatment of prediabetics is not as intensive as treating diabetics. Comorbidities increase the costs of both pharmacotherapy and specialised medical care compared to patients without complications. Earlier identification and treatment could improve the prediction of prediabetics and achieve cost savings.

# Abstract: Diabetes control in the community, under the management of a diabetes-clinic nurse specialist, and the effect of a parallel diabetologist advice

**Authors:** Joseph Azuri, Tzipi Gadot

**Affiliations:** Tel Aviv University and Maccabi Healthcare Services, Tel Aviv, Israel

**Keywords:** Nurse, diabetes, diabetologist

## Abstract

**Background:** Nurses play a key role in managing care and educating people with diabetes in various healthcare systems worldwide, while physicians play this role in others. In addition, some healthcare systems authorise specialist nurses to change patient dose regimens.

**Research question:** We examined diabetes outcomes in the community by the management of a diabetes-clinic nurse only versus parallel management with a diabetologist.

**Methods:** Retrospective data were collected for 100 consecutive people with diabetes registered in a community clinic with a diabetes-clinic nurse specialist as a case manager from 1/2018. About half of the patients selected received parallel advice from a diabetologist. Inclusion criteria included poor control of type 2 diabetes (HbA1c above 9%). Patients using an insulin pump, glucose sensor technologies, or multiple injection insulin programs were excluded.

**Results:** One hundred people with diabetes who met the study criteria were included, 64 males and a mean age of 60.03 ± 11.11. In a follow-up of 161.31 ± 68.65 days, HbA1c levels decreased by 3.17 ± 1.95% and did not change in a further follow-up of 162.36 ± 72.75 days. Significant improvement was also observed in controlling the other risk factors examined. Parallel consultation with a diabetologist and the nurse’s use of her authority for dose modifications showed no differences in all parameters. Logistic regression for analysing the effect of the variables related to the patient showed only a moderate effect.

**Conclusion:** Access to professional services remains a significant challenge for healthcare systems in long-term diabetes control. Monitoring poorly controlled people with diabetes with the help of a diabetes-clinic nurse specialist in the community clinic significantly improved diabetes and risk factors control and persisted long after the intervention. Healthcare systems should consider expanding the service of the diabetes-clinic nurse specialist in the community. Further studies will be required to examine the results in different patient subgroups.

# Abstract: Increased fracture risk among children and adolescents with coeliac disease: A nationwide cohort study

**Authors:** Galia Zacay, Ilana Weintrob, Dalit Modan-Moses, Yael Levy-Shraga

**Affiliations:** Meuhedet HMO, Ganei-Tikva, Israel

**Keywords:** Coeliac disease, paediatric fractures, cohort study, epidemiology

## Abstract

**Background:** One of the complications of coeliac disease (CD) is decreased bone mass. We aimed to analyse the risk of fractures among children with CD compared with matched children without CD; and to identify clinical and laboratory risk factors to fractures among children with CD.

**Research question:** The current study aimed to evaluate the fracture incidence rate of children with coeliac disease compared with a large, matched healthy population. A second aim was to identify clinical and laboratory risk factors for fractures among children with coeliac.

**Methods:** This registry-based cohort study included 2,372 children with CD and a large, matched comparison group of 11,860 children. Demographic and clinical data, anthropometric measurements and laboratory results were extracted from the electronic database of Meuhedet, a health maintenance organisation. Coded diagnoses identified fracture events at ages 1–18 years.

**Results:** The overall fracture incidence rate was 256 per 10,000 patient-years (PY) in the CD group and 165 per 10,000 PY in the comparison group (*p* < 0.001). Among boys, the fracture incidence rates were 301 per 10,000 PY and 202 per 10,000 PY (*p* < 0.001) for the respective groups and among girls 224 vs 138 per 10,000 PY, respectively (*p* < 0.001). The stratified hazard ratios (HR) to have a fracture was 1.57 (95%CI 1.43–1.73, *p* < 0.001) and for multiple fractures 1.67, (95%CI 1.38–2.01, *p* < 0.001). Analysis of the periods before and after the diagnosis of CD separately showed that during the period until the diagnosis, the stratified HR for fractures among children with CD compared to children without coeliac was 1.64 (95%CI 1.42–1.88, *p* < 0.001). The stratified HR from the diagnosis to the end of follow-up was 1.46 (95%CI 1.26–1.71, *p* < 0.001).

# Abstract: Lowering long-term use of benzodiazepine receptor agonists: A primary care perspective

**Authors:** Kristien Coteur, Sanne Peters, Pieter Jansen, Birgitte Schoenmakers, Marc Van Nuland

**Affiliations:** KU Leuven, Leuven, Belgium

**Keywords:** Primary care, benzodiazepines, insomnia, patients, general practitioners, pharmacists

## Abstract

**Background:** Long-term use of benzodiazepine receptor agonists (BZRA) is a global health issue. Its complications jeopardise the personal health of BZRA users and come with substantial socio-economic costs. Nevertheless, managing the decrease and stopping of BZRA use for insomnia in primary care remains difficult. Motives to use BZRA in the long term remain unclear and we currently have little direction on adequately supporting appropriate use.

**Research question:** Which factors contribute to long-term BZRA use for insomnia in primary care, from patients,’ general practitioners,’ and pharmacists’ perspectives?

**Methods:** Qualitative research with in-depth semi-structured interviews, following a grounded theory approach, set in primary care in Belgium. Twenty-four participants were interviewed, including nine patients, six general practitioners, and nine pharmacists. Transcripts were analysed using the Framework Method. Thematic findings were interpreted in the context of the Theoretical Domains Framework.

**Results:** We found a reflexive relation between views about hypnotic use at the level of society, healthcare, and patients. Six key messages captured factors that could contribute to lowering long-term BZRA use for insomnia in primary care: (1) societal beliefs as a game changer; (2) the opportunity of non-pharmacological treatment; (3) collaboration in primary care; (4) patient-centred goals; (5) informed consent and (6) self-management. Consistent with these factors, the participants discussed many ideas for interventions, mainly focused on the TDF domain of environmental context and resources.

**Conclusion:** Long-term BZRA use for insomnia is a multifaceted health problem that is not adequately addressed now. The current social and healthcare context are not empowering patients and professionals to lower long-term BZRA use for insomnia. Stakeholders have multiple ideas on how to turn the tide. Specifically, for primary care, all stakeholders reported needing a non-medicalised relationship between the patient and general practitioner to improve prescribing rates.
